# Integration of molecular docking and molecular dynamics simulations with subtractive proteomics approach to identify the novel drug targets and their inhibitors in *Streptococcus gallolyticus*

**DOI:** 10.1038/s41598-024-64769-z

**Published:** 2024-06-26

**Authors:** Peng Chao, Xueqin Zhang, Lei Zhang, Aiping Yang, Yong Wang, Xiaoyang Chen

**Affiliations:** 1 Department of Cardiology, People’s Hospital of Xinjiang Uygur Autonomous Region, Urumqi, China; 2 Department of Nephrology, People’s Hospital of Xinjiang Uygur Autonomous Region, Urumqi, China; 3 Department of Traditional Chinese Medicine, People’s Hospital of Xinjiang Uygur Autonomous Region, Urumqi, China

**Keywords:** *Streptococcus gallolyticus*, Endocarditis, Core proteomics, Glucosamine-1phosphate N-acetyltransferase (GlmU), RNA polymerase sigma factor (RpoD), Pantetheine-phosphate adenylyltransferase (PPAT), Computational biology and bioinformatics, Microbiology

## Abstract

*Streptococcus gallolyticus (Sg)* is a non-motile, gram-positive bacterium that causes infective endocarditis (inflammation of the heart lining). Because Sg has gained resistance to existing antibiotics and there is currently no drug available, developing effective anti-Sg drugs is critical. This study combined core proteomics with a subtractive proteomics technique to identify potential therapeutic targets for Sg. Several bioinformatics approaches were used to eliminate non-essential and human-specific homologous sequences from the bacterial proteome. Then, virulence, druggability, subcellular localization, and functional analyses were carried out to specify the participation of significant bacterial proteins in various cellular processes. The pathogen’s genome contained three druggable proteins, glucosamine-1phosphate N-acetyltransferase (GlmU), RNA polymerase sigma factor (RpoD), and pantetheine-phosphate adenylyltransferase (PPAT) which could serve as effective targets for developing novel drugs. 3D structures of target protein were modeled through Swiss Model. A natural product library containing 10,000 molecules from the LOTUS database was docked against therapeutic target proteins. Following an evaluation of the docking results using the glide gscore, the top 10 compounds docked against each protein receptor were chosen. LTS001632, LTS0243441, and LTS0236112 were the compounds that exhibited the highest binding affinities against GlmU, PPAT, and RpoD, respectively, among the compounds that were chosen. To augment the docking data, molecular dynamics simulations and MM-GBSA binding free energy were also utilized. More in-vitro research is necessary to transform these possible inhibitors into therapeutic drugs, though computer validations were employed in this study. This combination of computational techniques paves the way for targeted antibiotic development, which addresses the critical need for new therapeutic strategies against *S. gallolyticus* infections.

## Introduction

Formerly known as *Streptococcus bovis*, *Streptococcus gallolyticus* (Sg) is a gram-positive, non-motile bacterium. This strain of Lancefield Group D Streptococci bacteria has a variety of phenotypes^1^. Despite its frequent presence in microflora, 2.5–15% of it is found in the digestive tract of a healthy individual. It then becomes an opportunistic pathogen that causes a variety of diseases, including infecting endocarditis, meningitis, colon cancer, and septicemia^2^.

Over the past 20 years, the incidence of infectious endocarditis has rapidly grown worldwide^3–5^. The 2.6–7 cases of endocarditis per 100,000 individuals reported annually were primarily caused by streptococcal infections; incidences were 39% in South America, 31% in other European countries, 17% in North America, and 32% worldwide^5^. With a median age of 58, the disease primarily affects older adults^6^. Sg endocarditis is more common in people who eat dairy products, have a history of hepatic disease, and have conditions like diabetes mellitus and rheumatoid arthritis^7^.

Sg tries to harm the endocardium when a metabolic disorder, a primary infection, or a compromised immune system are present. A thrombus develops as a result of this injury due to the loss of platelets and fibrin. The germs enter the bloodstream through the thrombus after thrombus development. Because of its virulence traits, Sg can enter the bloodstream paracellularly and adhere to the endocardium—the collagen-rich surface of the damaged heart valve—without triggering a strong immune reaction. After sticking to the endocardium, this bacterium grows and creates a biofilm, inflaming the heart's lining and causing endocarditis^8,9^.

Gentamycin, Penicillin G, and streptomycin are frequently recommended antibiotics for infected endocarditis. Vancomycin and gentamicin-related ceftriaxone are alternative options for those who are allergic to penicillin^10^. For individuals with a prolonged fever who are not responding to medication, an expensive surgical procedure may be necessary. Sg is penicillin-resistant, and one strain has tetracycline resistance^11^. Hence, an effective endocarditis treatment strategy, novel therapeutic targets, and an effective drug are urgently required.

Numerous computer techniques for quick identification have been created, including subtractive genomics and core genomics, which enable us to find the key genes that are essential but not identical to the human genome^12^. Molecular docking and molecular dynamics (MD) simulations are important in drug discovery because they can provide detailed information about the interactions between potential drug candidates and biological targets. Molecular docking predicts the preferred orientation and binding affinity of small molecules (ligands) to target proteins, assisting in the identification of promising candidates through simulation and scoring of their interactions in the binding site. MD simulations, on the other hand, provide a time-dependent view of these interactions, revealing the ligand-protein complex's stability, conformational changes, and potential allosteric effects. These techniques work together to improve molecular mechanism understanding, lead compound optimization, and the efficiency and accuracy of the drug development pipeline^1^. The aim of this research is to utilize in silico methodologies to identify putative therapeutic targets through the integration of the Sg species' genetic and proteome information.

## Material and methods

### Core proteomes identification

All Sg strains with full assembly levels were taken into account for pan-genome analysis in this investigation. Hosts were thought to be humans. We selected seven distinct strains of Sg and downloaded their proteomes from NCBI. Additional File 1: Table S1 contains their information. The Bacterial Pan Genome Analysis Tool (BPGA) was then used to conduct a core proteome analysis on the proteomes in order to find conserved proteins that are shared by all strains and identify possible therapeutic candidates^13^.

### Identification of essential proteins

It is thought that essential proteins are necessary for the survival of cells. In order to identify essential proteins, the core proteome of Sg strains was uploaded to the Geptop 2.0 server. Essentiality was defined as 0.24 as the cutoff value. Geptop is applicable to all species of bacteria that possess a sequenced genome^14,15^.

### Non-homologous proteins identification

In order to detect non-homologous protein sequences of Sg, BlastP was used to analyze essential proteins against the human proteome using default parameters^16^. Non-homologous proteins are those that have no resemblance to the human proteome.

### Subcellular localization prediction

Predicting protein subcellular localization is critical for genome annotation and genome analysis in bacterial infections because these proteins may be the primary targets for drugs or vaccines^17^. Non-homologous protein subcellular localization was predicted using the BUSCA server^18^.

### Virulent proteins identification

Additionally, the chosen proteins were run using BLASTp against the Virulence Factor Database using an E-value cut-off of 10^−5^ to determine which proteins had the highest virulence factor^19^.

### Druggability of essential proteins

Later, virulent proteins were compared to FDA-approved therapeutic proteins obtained from the DrugBank using BLASTp^20^. The identification of drug-target-like properties of identified essential proteins and the prioritization of innovative and distinctive therapeutic targets were accomplished using the E-value cut-off of 10^−5^. We compared the query protein sequence to the protein sequences in DrugBank using BLASTp. This allowed us to identify proteins in DrugBank that have a significant sequence similarity to our query protein. Identifying these similar proteins allowed us to predict potential interactions and therapeutic applications based on existing drug-target relationships in DrugBank. This method uses known protein targets to find promising candidates for drug repurposing or new drug-target interactions.

### Functional prediction

The development of novel drugs depends heavily on our understanding of biological mechanisms, molecular functions, and protein structural characteristics. Biological processes and molecular activities of the target proteins were predicted by the InterProScan server^21^.

### Homology modeling and structure prediction

The shortlisted proteins from subtractive genomics were examined, and the Protein Data Bank (PDB) was searched for their structures^22^. To discover an appropriate template for protein structure modeling, BLASTp was employed. The protein structure was modeled using the Swiss Model-Homology Modeler because there is no 3-dimensional structure^23^. The most accurate and effective way to create 3D protein structures in the absence of an empirically known protein crystal structure is through homology modeling. It compares protein sequences in the Protein Data Bank..

### Validation of protein structures

The modelled structures were validated using a range of techniques based on their respective concepts. i.e. PROCHECK is used to evaluate the stereochemistry composition of a protein structure by examining both the protein’s overall structural geometries and its residue-by-residue geometry. The ERRAT, or empirical atom-based analysis tool, and the PROSA web service, which use Z-score to compare the modeled protein structure to the PDB-supplied structures^24–26^.

### Ligand preparation

The library of natural products containing 1400 compounds were obtained from the LOTUS database (https://lotus.naturalproducts.net/) which is a repository of natural products. The ligand structures were prepared for additional research using the LigPrep program from Schrödinger’s Maestro software suite^27^. For every ligand, LigPrep is used to optimize geometries and produce conformers. To accommodate their different orientations and possible flexibility, 32 conformers were constructed for every ligand in this case. The OPLS_2005 forcefield was utilized to modify the ligands’ geometry in order to guarantee that they were in conformations that were energetically favorable. A popular empirical forcefield for displaying and computing the energy of atom-to-atom interactions in molecules is the OPLS_2005 forcefield^28^. Lowering the compounds' energy eliminated any unfavorable interactions or strained geometries, producing more dependable structures for upcoming studies.

### Protein preparation

This study aims to clarify the virtual screening process of natural products against Sg target proteins and identify the best hits. The protein structures were prepared for further study using the Protein Preparation Wizard in the Schrödinger Maestro software suite^29^. The process of preparing the protein involved multiple steps. Disulfide bonds were created, bond orders were set, and zero-order metal bonds were allocated. Hydrogens were also introduced into the protein structures. In the crystal structures, all unnecessary water molecules and ligands were eliminated. Using the PROPKA program, the pKa values of the ionizable groups in the proteins were determined, allowing the hydrogen bond network of the proteins to be optimized at pH 7.0^30^. Lastly, an empirical forcefield that is widely used for molecular simulations, the OPLS_2005 forcefield, was used to reduce the energy of the protein structures. After protein synthesis, all receptors were found to have 3D grids built at specific reported sites. Applying the site-specific docking technique to these grids allowed researchers to examine the binding patterns and interactions of ligands deposited into pre-specified binding sites on proteins.

### Molecular docking

The Glide docking module was utilized in SP (Standard Precision) mode to dock the prepared ligands at designated locations on the prepared protein structures^31^. Based on their glide scores, the docked ligands were assessed and selected.

### ADMET analysis

To determine the physicochemical and ADMET (absorption, distribution, metabolism, excretion, and toxicity) properties of the docked ligands, additional analysis was conducted. Maestro’s QikProp tool was used to predict these attributes^32^. Depending on the ligands’ molecular structures, the QikProp tool estimates multiple attributes. QPPCaco, QPlogBB, hydrogen bond acceptors, QPlogHERG, molecular weight, hydrogen bond donors, QPlogPo/w, and QPlogKhsa are typical expected features. The size and complexity of a ligand are indicated by its molecular weight. The count of hydrogen atoms and atom centers that are potentially involved in hydrogen bonding interactions is determined by hydrogen bond donors and acceptors, respectively. Membrane permeability and the compound’s hydrophobicity are assessed by the octanol-water partition coefficient logarithm predicted by QPlogPo/w. QPlogHERG determines whether a ligand may be blocking the hERG potassium channel, which is associated with potential cardiac toxicity. QPPCaco calculates compound permeability by modeling intestinal absorption using the Caco-2 cell monolayer. In order to estimate a compound's ability to penetrate the blood-brain barrier and enter the central nervous system, QPlogBB calculates the logarithm of the BBB partition coefficient. The human serum albumin protein, which is essential for drug binding and bloodstream distribution, is used by QPlogKhsa to calculate the logarithm of the binding affinity.

### MD simulation

MD simulations of a few selected compounds were run for 100 ns using Desmond^33^. The protein-ligand complexes’ stability was investigated using MD simulations. Preprocessing, optimization, and minimization were performed on the complexes to prepare them for simulation. The force field OPLS_2005 was employed to minimize the complexes. An orthorhombic box of (size Å × 10 Å × 10 Å) TIP3P water molecules was used to solvate the systems^34^. The systems were neutralized by introducing 0.15 M NaCl salt and counter ions as necessary to replicate physiological conditions. The NPT ensemble was calibrated to operate at 1 atm of pressure and 300 K of temperature. Before the simulation started, the systems were given some time to unwind. Throughout the simulation, trajectories were recorded and stored at 40 ps intervals to enable further analysis of the outcomes.

## Results

### Prioritizing drug candidates and retrieving core proteomes

Seven well-known pathogenic strains of Sg were considered for this study. The core proteome was analyzed, and 9576 proteins were identified. 325 out of 9576 proteins had been identified as essential genes after being uploaded to the Geptop server. An essential protein that is not human-like was discovered using Blast P. Of the 325 essential proteins, 138 were found to be non-homologous. It was possible to ascertain how specific proteins carry out their functions by using the prediction of subcellular localization. It was previously believed that proteins found in the cytoplasm could serve as therapeutic targets^2,3^. Hence, 109 cytoplasmic proteins were screened from 138 non-homologous proteins. Further, a BLASTp investigation against the Database of Virulence factor was used to assess these 109 proteins. Only 12 of these, or proteins with a high virulence factor for Sg, were categorized as essential virulent proteins. The BLASTp examination against the Drug Bank Database was also performed on these 12 virulent proteins. Only those proteins that share a significant amount of sequence similarity with FDA-approved therapeutic targets were picked, and the remaining ones were excluded. The entire drug development process depends on having a thorough understanding of biological mechanisms, molecular functions, and the specifics of protein structure. It aids not just in the discovery of prospective therapeutic targets but also in the development, testing, and optimization of drugs used to treat a variety of diseases. The InterProScan functional prediction results were used to assess three proteins as possible therapeutic targets. Information about these three pharmacological targets can be found in Table 1.

**Table 1:** Functional details of selected drug targets.

Proteins name	Molecular function	Biological processes
Glucosamine-1phosphate N-acetyltransferase (GlmU)	Magnesium ion binding	Cell morphogenesis
	UDP-N-acetylglucosamine diphosphorylase activity	UDP-N-acetylglucosamine biosynthetic process
	Glucosamine-1-phosphate N-acetyltransferase activity	Peptidoglycan biosynthetic process
RNA polymerase sigma factor (RpoD)	Sigma factor activity	DNA-templated transcription initiation
	DNA-binding transcription factor activity	Regulation of DNA-templated transcription
	DNA binding	
Pantetheine-phosphate adenylyltransferase (PPAT)	Pantetheine-phosphate adenylyltransferase activity	Coenzyme A biosynthetic process
	Catalytic activity	Biosynthetic process

### Structure prediction and validation

**Figure 1:**
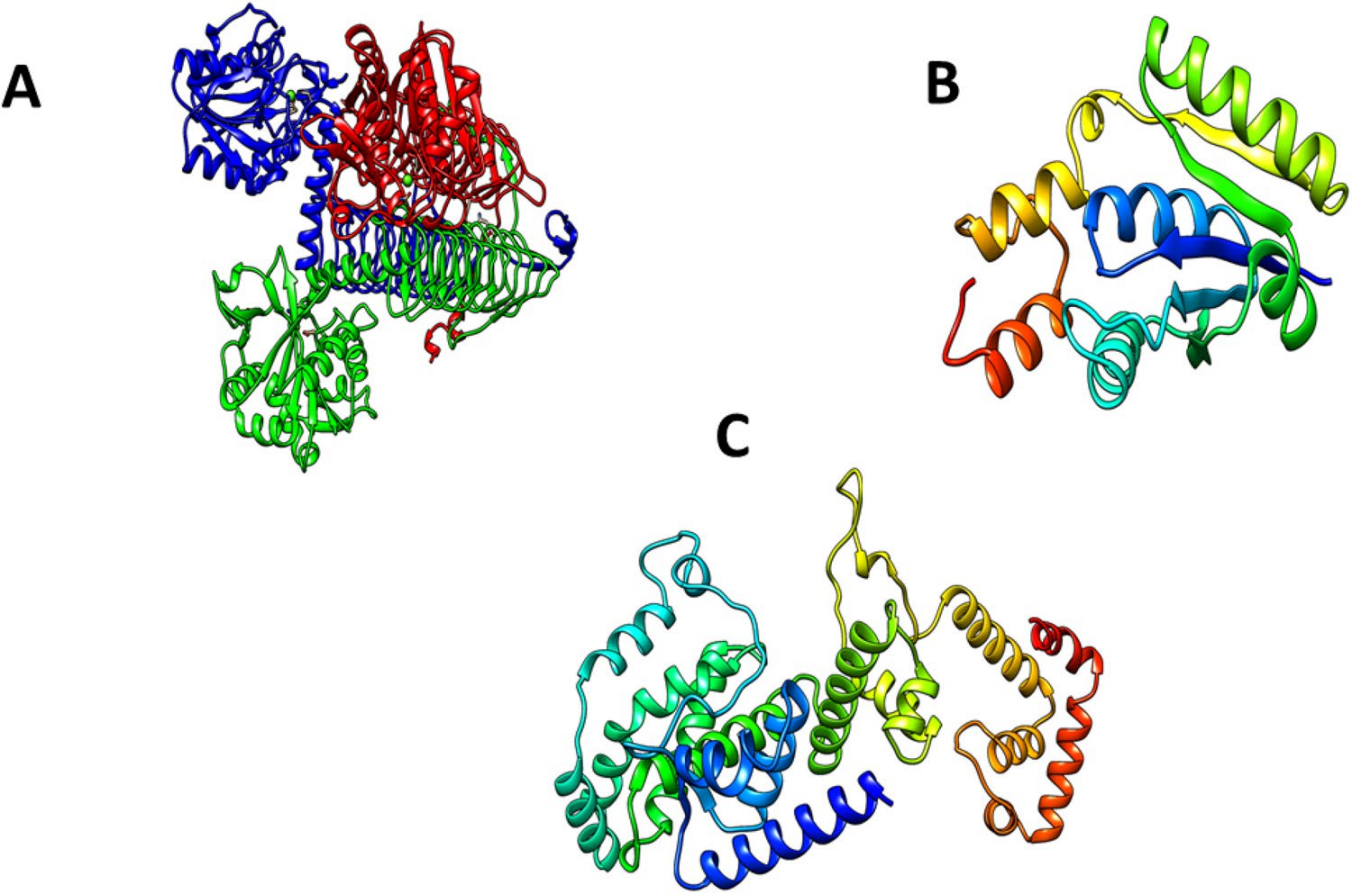
3D structures of the target protein. (**A**) GlmU (**B**) PPAT (**C**) RpoD.

The FASTA sequences of proteins from the NCBI database were utilized for homology modeling. Swiss Model was used to model the structures (Fig. 1). Table 2 enlists the detail of the modeled structures.

**Table 2:** Details of the model structure.

Protein accession no	Template used	Sequence identity (%)	Query coverage (%)	GMQE
GlmU (WP_058621458.1)	1hm9.2.C	76.47	100	0.91
RpoD (WP_114318613.1)	A0A380KGI9.1.A	96.48	100	0.83
PPAT (WP_061458921.1)	A0A368UEH5.1.A	97.58	100	0.94

**Figure 2:**
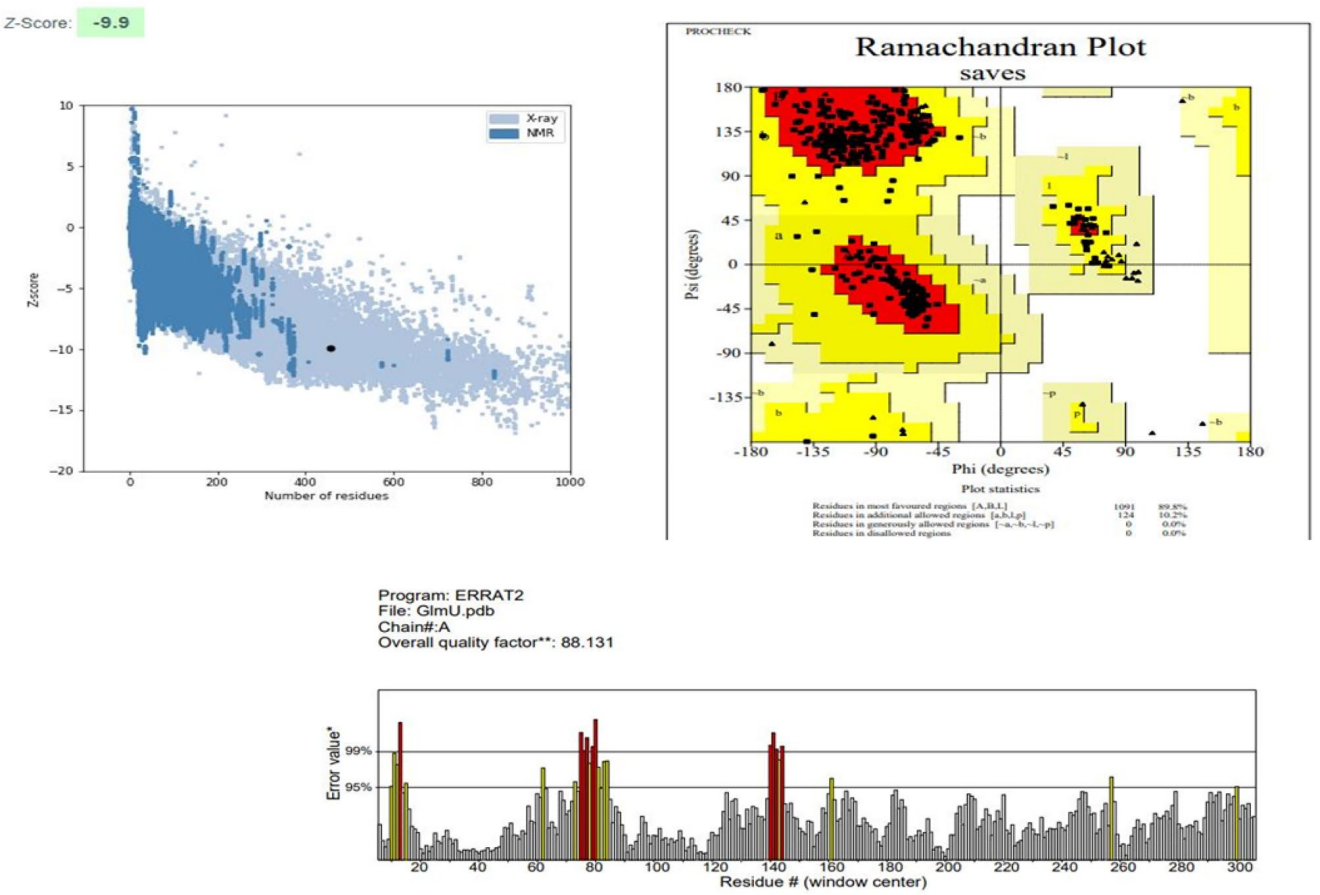
Evaluation details of the GImU structure.

**Figure 3:**
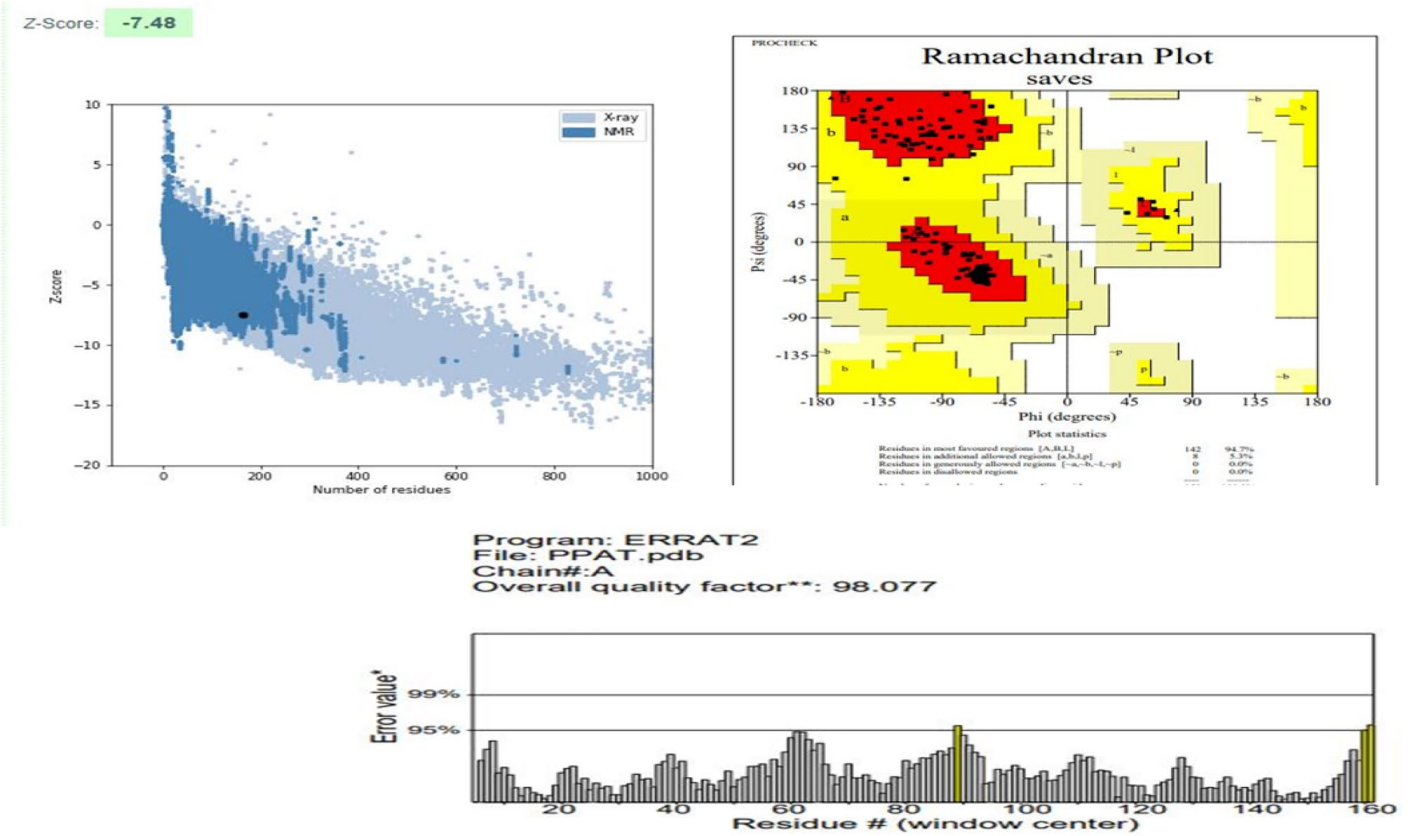
Evaluation details of the PPAT structure.

**Figure 4:**
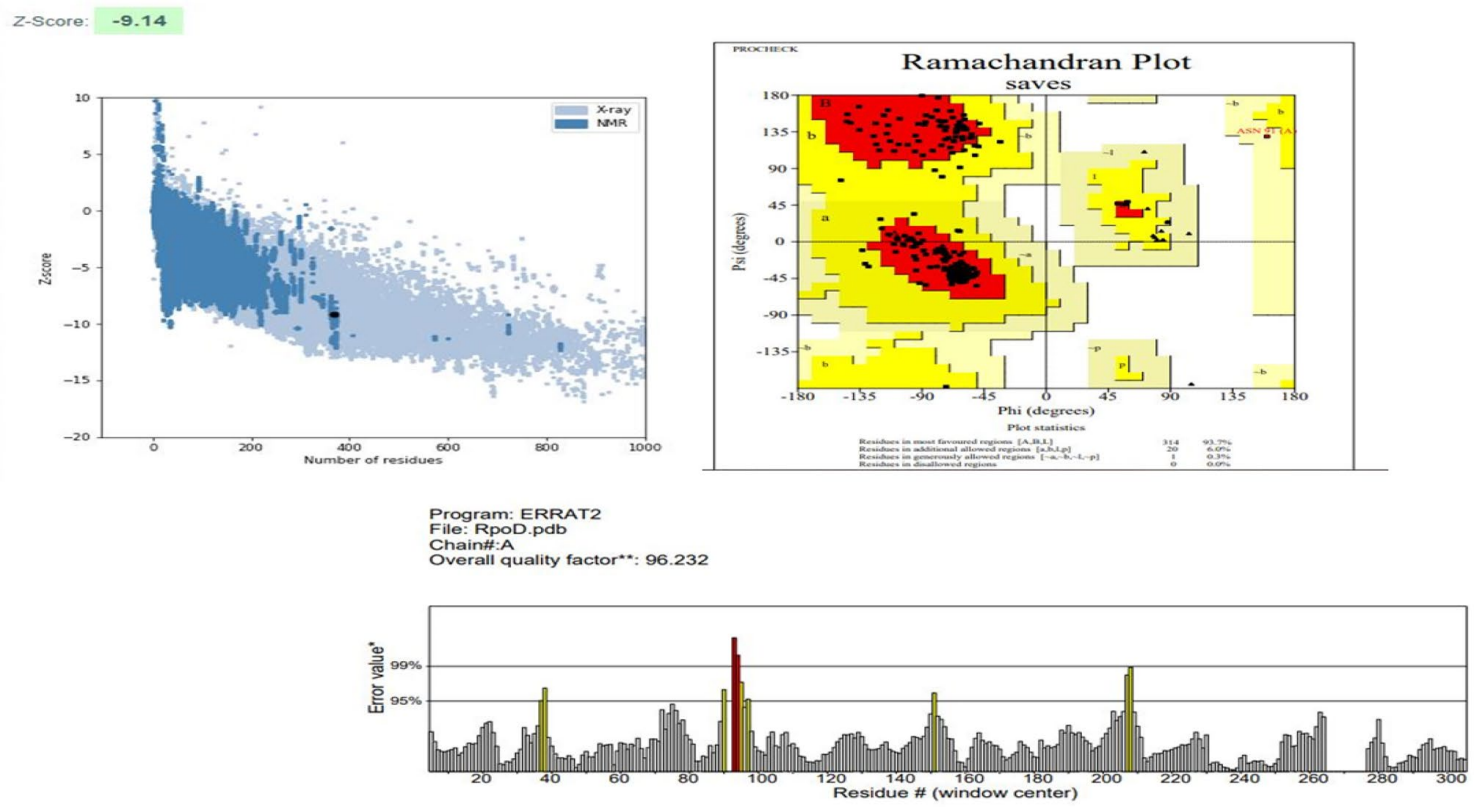
Evaluation details of the RpoD structure.

Predicted models were than evaluated by PROCHECK, PROSA web and ERRAT server. The results of these servers indicated the good quality of the structures. Figure 2 represents the evaluation details of the GImU structure. A Z-score of −9.9 indicates that the GImU structure falls within the range of scores typically found in high-quality native protein structures, implying that the model is likely to be reliable. A quality factor of 88.1 indicates that 88.1% of the protein model’s volume is error-free, which is considered excellent and suggests a well-refined and accurate model. Similarly, in the Ramachandran plot, having 89.8% of residues in favored regions indicates that the majority of residues’ backbone conformations are in energetically favorable regions. This high percentage indicates that the protein model has good stereochemical quality and is properly folded. The PPAT structure is of high quality, as indicated by a PROSAweb Z-score of −7.48, showing good alignment with known protein structures; a 98.0 quality factor on the ERRAT server, reflecting excellent non-bonded atomic interaction accuracy; and 94.7% of residues in the favored region of the Ramachandran plot, suggesting proper stereochemical conformation and reliable modeling (Fig. 3). Similarly, the RpoD structure is of high quality, as evidenced by a PROSAweb Z-score of −9.14; a 96.2% quality factor on the ERRAT server,; and 93.7% of residues in the favored region of the Ramachandran plot (Fig. 4).

### Molecular docking

Employing the glide tool’s conventional precision protocol, the library of natural products comprising 1400 compounds that were retrieved from the LOTUS database was docked against GlmU, PPAT, and RpoD. The top 10 compounds that docked against each target protein were selected after the docking results were evaluated using the glide gscore (Table 3). In GlmU docking analysis, the selected natural products showed affinities in the range of −9.263 to −8.622 kcal/mol. For PPAT, the docking scores of the selected products were −8.038 to −7.25 kcal/mol. In the RpoD, the hits showed the binding affinities ranging from −7.154 to −6.521. Docking results were further validated by MD simulation and MMGBSA analysis.

**Table 3:** List of compounds virtually screen based on their interactions score.

No	Compounds	Structures	Glide score
GlmU			
1	LTS0001632		−9.263
2	LTS0264141		−9.051
3	LTS0045589		−8.985
4	LTS0006448		−8.947
5	LTS0059100		−8.793
6	LTS0178253		−8.782
7	LTS0033334		−8.711
8	LTS0171683		−8.708
9	LTS0094833		−8.691
10	LTS0139202		−8.622
PPAT			
1	LTS0243441		−8.038
2	LTS0108229		−7.899
3	LTS0216732		−7.879
4	LTS0257496		−7.746
5	LTS0030191		−7.511
6	LTS0228021		−7.511
7	LTS0068514		−7.433
8	LTS0013471		−7.364
9	LTS0212684		−7.306
10	LTS0064984		−7.255
RpoD			
1	LTS0236112		−7.154
2	LTS0065921		−7.106
3	LTS0159945		−7.104
4	LTS0203029		−6.833
5	LTS0157950		−6.758
6	LTS0275484		−6.705
7	LTS0261418		−6.66
8	LTS0256777		−6.659
9	LTS0157665		−6.563
10	LTS0216052		−6.521

### ADMET analysis

The physicochemical and ADMET characteristics of the chosen hits were also examined. A criterion for drug-likeness called Lipinski's rule of five was violated in one or two cases by most of the compounds i.e., the molecular weight of most of the compounds was more than 500, similarly the hydrogen bond acceptors were greater than 10 and hydrogen bond donors were more than 5. The compounds, however, fell within the permitted ranges for the ADMET attributes. “QPlogPo/w” (−2.0 to 6.5), “QPPCaco” (< 25 poor, > 500 great), “QPlogHERG” (< −5), “QPlogBB” (−3.0 to 1.2), and “QPlogKhsa” (−1.5 to 1.5) were the cutoff values for the ADMET parameters. Table 4 lists the compounds' physicochemical and ADMET properties.

**Table 4:** The ADMET and physicochemical characteristics of the chosen compounds as determined by QikProp.

Compounds	MW	HBD	HBA	QPlogPo/w	QPlogHERG	QPPCaco	QPlogBB	QPlogKhsa
GlmU								
LTS0271681	523.493	6	18	−0.913	−5.077	30.558	−2.786	−1.183
LTS0185946	522.505	6	15	0.139	−3.758	1.835	−3.627	−0.968
LTS0008706	523.493	6	18	−1.331	−4.832	16.874	−3.005	−1.238
LTS0029004	457.436	5	13	0.171	−6.739	8.715	−3.781	−0.791
LTS0236079	526.582	4	13	2.011	−5.105	65.624	−2.447	−0.191
LTS0150761	474.42	3	11	0.703	−5.957	5.378	−3.565	−0.308
LTS0092860	504.49	6	13	0.013	−5.717	4.426	−4.022	−0.739
LTS0261418	522.505	6	17	−0.317	−6.368	17.314	−3.477	−1.059
LTS0044200	536.532	6	19	−0.64	−6.235	22.341	−3.21	−1.204
LTS0242752	462.452	5	13	0.482	−5.33	30.838	−2.812	−0.655
PPAT								
LTS0263188	456.489	6	13	0.151	−5.84	26.278	−3.269	−0.82
LTS0044020	528.468	6	13	0.242	−7.117	2.298	−4.704	−0.618
LTS0138804	538.504	8	16	−1.204	−6.959	1.134	−5.716	−1.263
LTS0151403	478.452	5	12	0.171	−5.84	26.278	−3.269	−0.655
LTS0165780	496.467	6	15	−0.534	−4.341	0.349	−4.462	−1.001
LTS0196533	506.419	6	14	−0.979	−2.385	2.669	−2.857	−0.776
LTS0155227	510.496	6	11	1.54	−5.602	1.315	−4.383	−0.36
LTS0090062	532.457	5	14	0.099	−7.729	11.282	−4.017	−0.874
LTS0153718	510.496	6	11	1.477	−4.402	0.345	−4.623	−0.401
LTS0150030	537.519	7	18	−0.938	−7.007	20.776	−3.395	−1.261
RpoD								
LTS0024216	536.534	6	11	2.118	−7.986	7.877	−4.438	−0.196
LTS0142659	496.467	7	16	−1.058	−6.446	3.89	−4.577	−1.174
LTS0257496	524.521	6	16	−0.615	−5.207	11.972	−3.45	−1.006
LTS0178696	502.431	4	13	0.334	−4.585	0.59	−4.231	−0.812
LTS0261807	504.446	7	13	−0.358	−6.919	1.569	−5.117	−0.851
LTS0186341	516.457	3	13	1.249	−4.967	2.464	−3.781	−0.711
LTS0015626	508.521	5	14	1.437	−2.609	0.541	−3.906	−0.986
LTS0074794	536.534	6	11	2.144	−7.851	11.837	−4.136	−0.227
LTS0033334	538.504	7	17	−0.801	−4.601	0.42	−4.895	−1.256
LTS0069049	504.49	6	13	0.225	−6.665	2.732	−4.885	−0.69

### Binding pose analysis

The study examined the molecular associations between natural product compounds and protein targets encompassing GlmU, PPAT, and RpoD. Based on the glide scores, the compound exhibiting the highest potential for each target was selected and subsequently subjected to further analysis to investigate its molecular interactions and the durability of its binding interactions.

### GlmU

**Figure 5:**
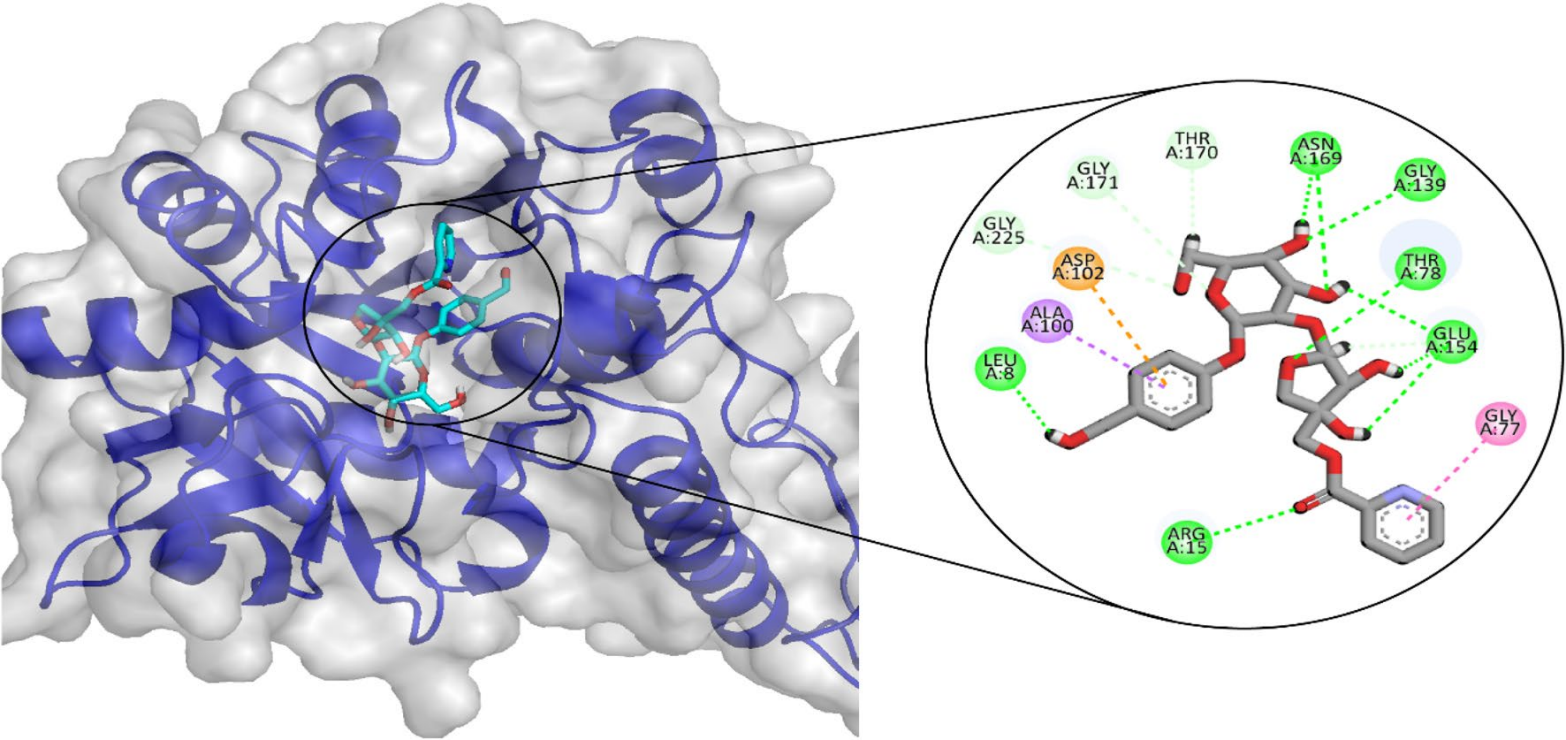
The molecular interactions of LTS0001632 against GlmU. The green spheres (hydrogen bonds) and the magenta spheres (hydrophobic interactions) represent the molecular interactions.

Of the natural product compounds, LTS0001632 showed the highest binding affinity against GlmU. After analyzing the molecular interactions, it was found that the compound formed six hydrogen bonds with Asn169, Glu154, Gly139, Thr78, Leu8, and Arg15. It also made three hydrophobic interactions as shown in Fig. 5.

**Figure 6:**
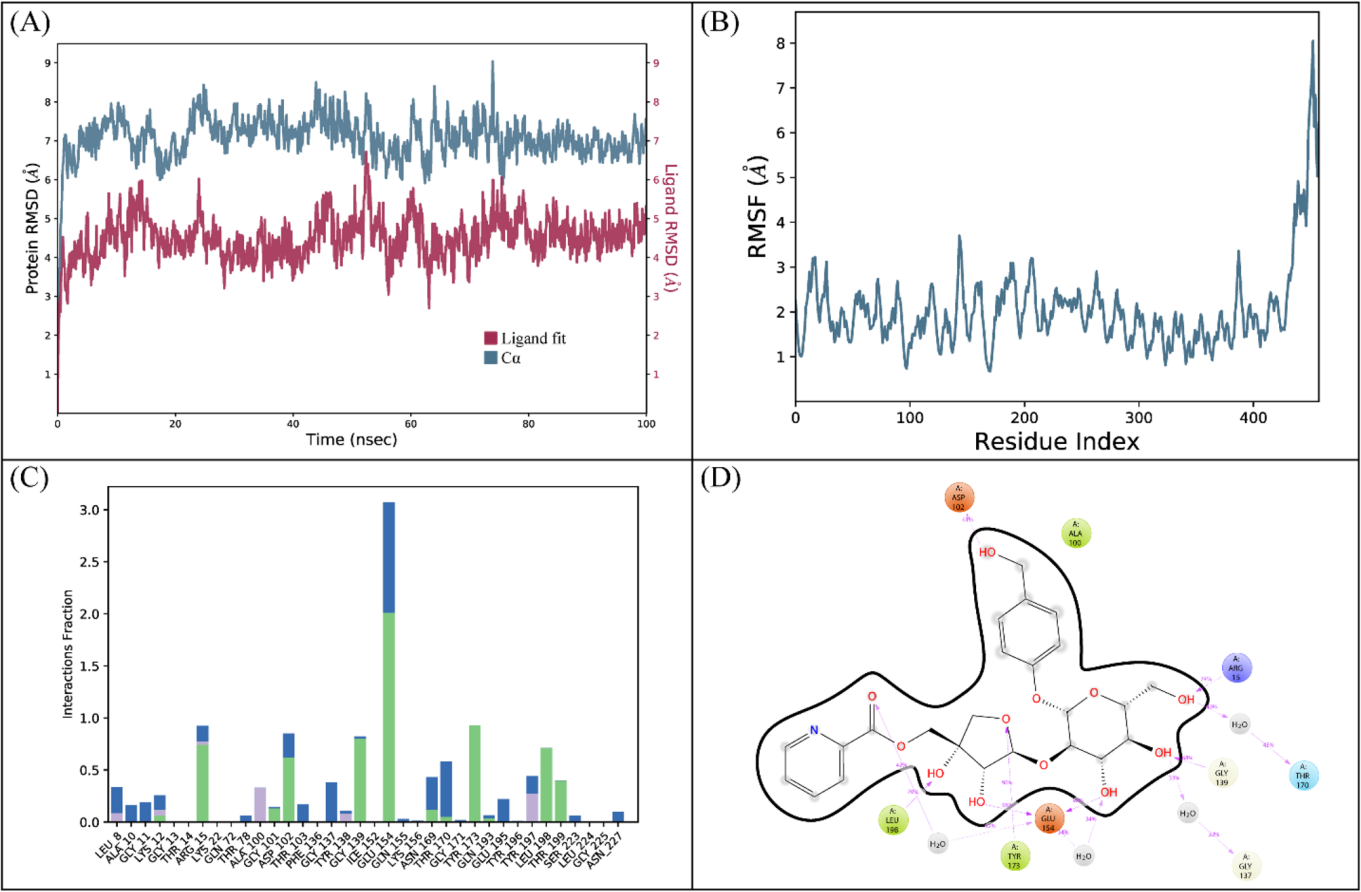
The MD trajectory analysis of the GlmU complex. (**A**) RMSD of C-alpha atoms of proteins and ligand atoms fit on protein. (**B**) RMSF analysis. (**C**) Protein-ligand contacts were calculated during the simulation. (**D**) Tendency of the interacting residues with the ligand during simulation.

A 100 ns simulation was performed to evaluate the complex’s stability. The RMSD of the protein’s C-alpha atoms and the ligand fit on the protein were used to calculate the stability of the complex. Throughout the simulation, the C-alpha atoms of the protein were observed to maintain their RMSD values within the range of ~ 7–8 Å, the large deviation in the RMSD arose due to the presence of large loop towards the C-terminal. But the protein structure attained stability after equilibration and did not show the deviation more than 2 Å which showed the stable configuration of protein, whereas the ligand's RMSD remained lower than that of the protein atoms (Fig. 6A). The structural dynamics of the protein residues were investigated by calculating the RMSF values, which show the flexibility of protein residues in response to ligand binding during simulation. Higher RMSF values suggest flexibility, while lower values indicate rigidity of the residues. The majority of the residues stayed rigid throughout the simulation, with the exception of the loop sections, where the RMSF values were ~ 4 Å (Fig. 6B). Ionic, hydrogen, and hydrophobic interactions were found to be the most important types of interactions between the ligands and the protein during an MD simulation study. These interactions are essential for regulating and stabilizing the functional characteristics of the protein-ligand complex. Hydrogen bonds were specifically formed by the residues Arg15, Gly101, Asp102, Gly139, Glu154, Tyr173, Leu198, and Thr199. There was no residue involved in the ionic interactions (Fig. 6C). The residue that showed the strongest propensity for binding among these interactions was Glu154, with interactions being seen in 96% of the total frames (Fig. 6D).

Additionally, the prime-MMGBSA module estimated the complex’s binding free energy. The sum of the Coulomb, Van der Waals, Solvation, and Covalent energies was the binding free energy. The van der Waals energy contribution was −60.24, solvation was 42.86, covalent energy was 11.05, coulombic energy was −38.54, and total binding free energy of the complex was −83.52 kcal/mol as shown in Table 5.

**Table 5:** The contribution of the energy components in total binding free energy in GlmU complex.

Components	Binding energy (kcal/mol)
Bind	−83.52
Coulomb	−38.54
Covalent	11.05
Solv GB	42.86
vdW	−60.24

### PPAT

**Figure 7:**
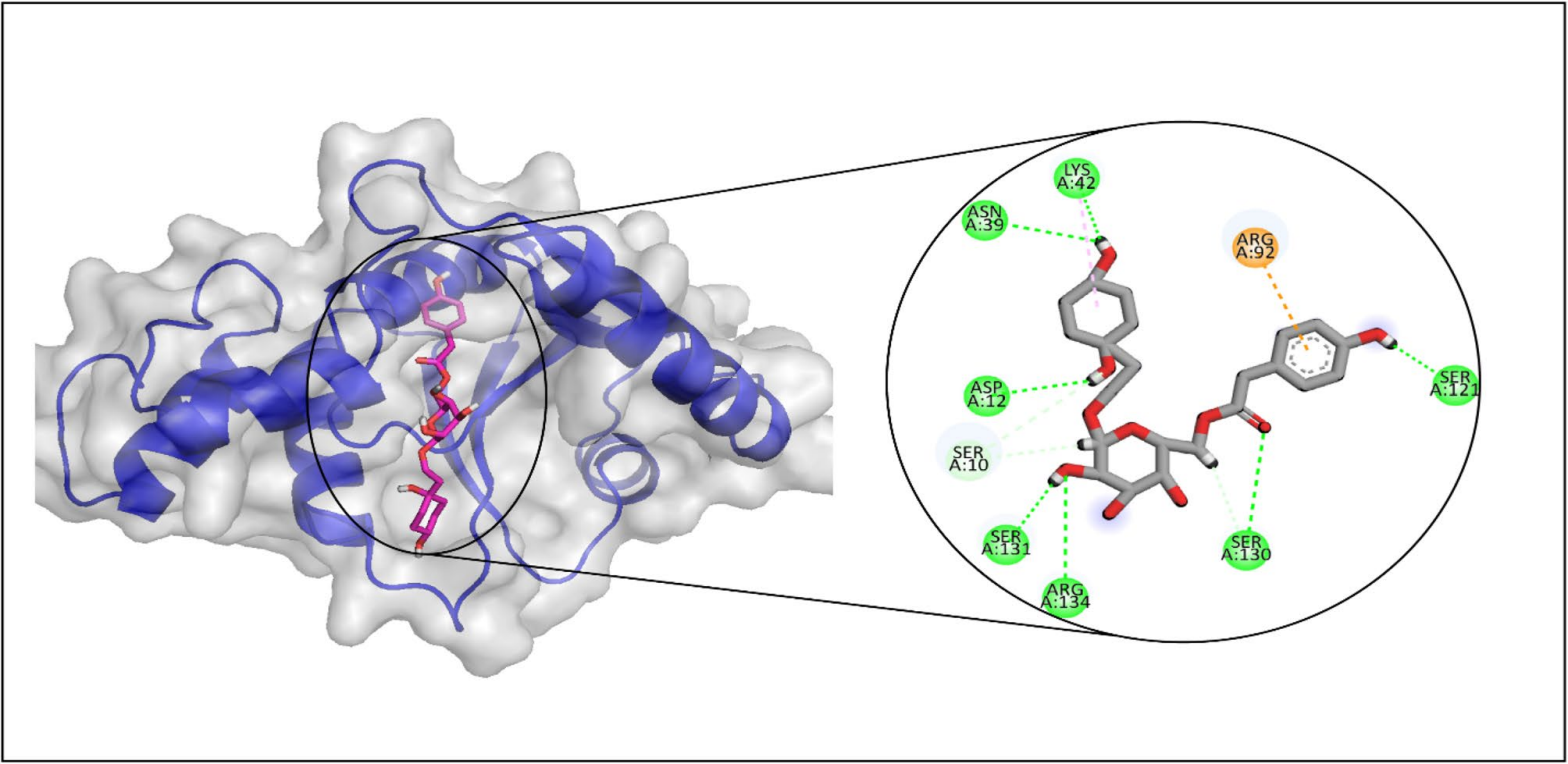
The molecular interactions of LTS0243441 against PPAT. The magenta spheres (hydrophobic/pi-alkyl) and the green spheres (hydrogen bonds) represent the molecular interactions.

The docking analysis of PPAT revealed that LTS0243441 showed the highest binding affinity among the selected ligands, so it was selected for the molecular interaction and stability analysis. The molecular interactions showed that LTS0243441 made hydrogen bonds with seven residues Lys42, Asn39, Asp12, Ser131, Arg134, Ser130, and Ser121. It also made one hydrophobic interaction with Lys42 as shown in Fig. 7.

**Figure 8:**
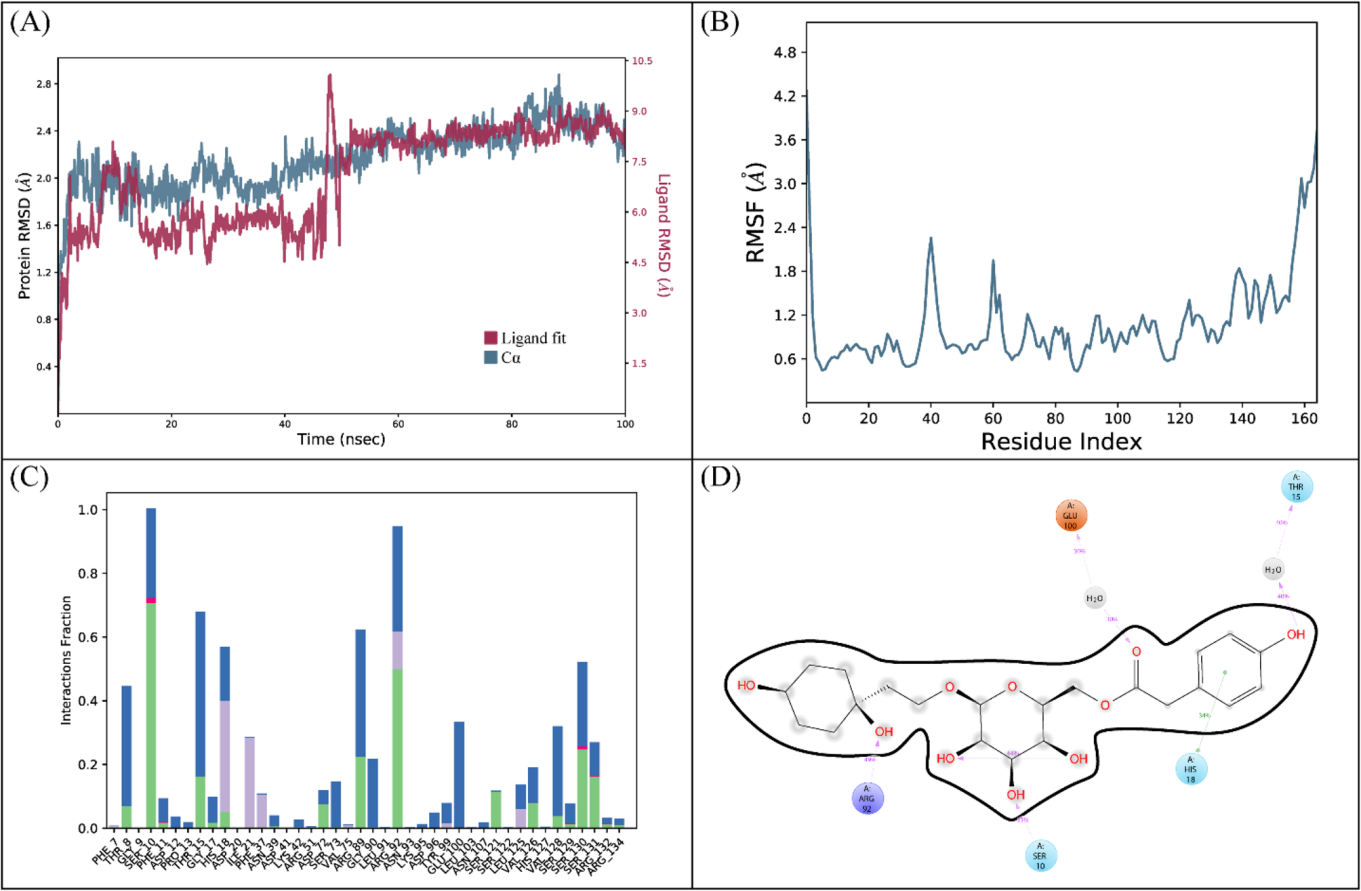
The MD trajectory analysis of the PPAT complex. (**A**) RMSD of C-alpha atoms of proteins and ligand atoms fit on protein. (**B**) RMSF analysis. (**C**) Protein-ligand contacts calculated during simulation. (**D**) Tendency of the interacting residues with the ligand during simulation.

The RMSD of C-alpha atoms of protein maintained the RMSD values in the range of ~ 2–2.4 Å after attaining equilibrium at 5 ns, and the RMSD of ligand was well fitted on the protein atoms which indicate the stability of the complex (Fig. 8A). The RMSF analysis showed that that most of the residues remained rigid during the simulation except for a loop region whose values reached ~ 2.4 Å (Fig. 8B). The specific residues involved in hydrogen bonding in protein-ligand contact analysis were Asp72, Arg89, Arg92, Ser121, Val126, Val128, Ser130, and Ser131 (Fig. 8C). With interactions seen in 49% of the total frames, Arg92 showed the strongest propensity for binding among these interacting residues (Fig. 8D).

The binding free energy calculation showed that the total binding free energy of the complex was −67.66 kcal/mol. The other energy components are shown in Table 6.

**Table 6:** The contribution of the energy components in total binding free energy in PPAT complex.

Components	Binding energy (kcal/mol)
Bind	−67.66
Coulomb	−46.30
Covalent	11.17
Solv GB	36.27
vdW	−37.06

### RpoD

**Figure 9:**
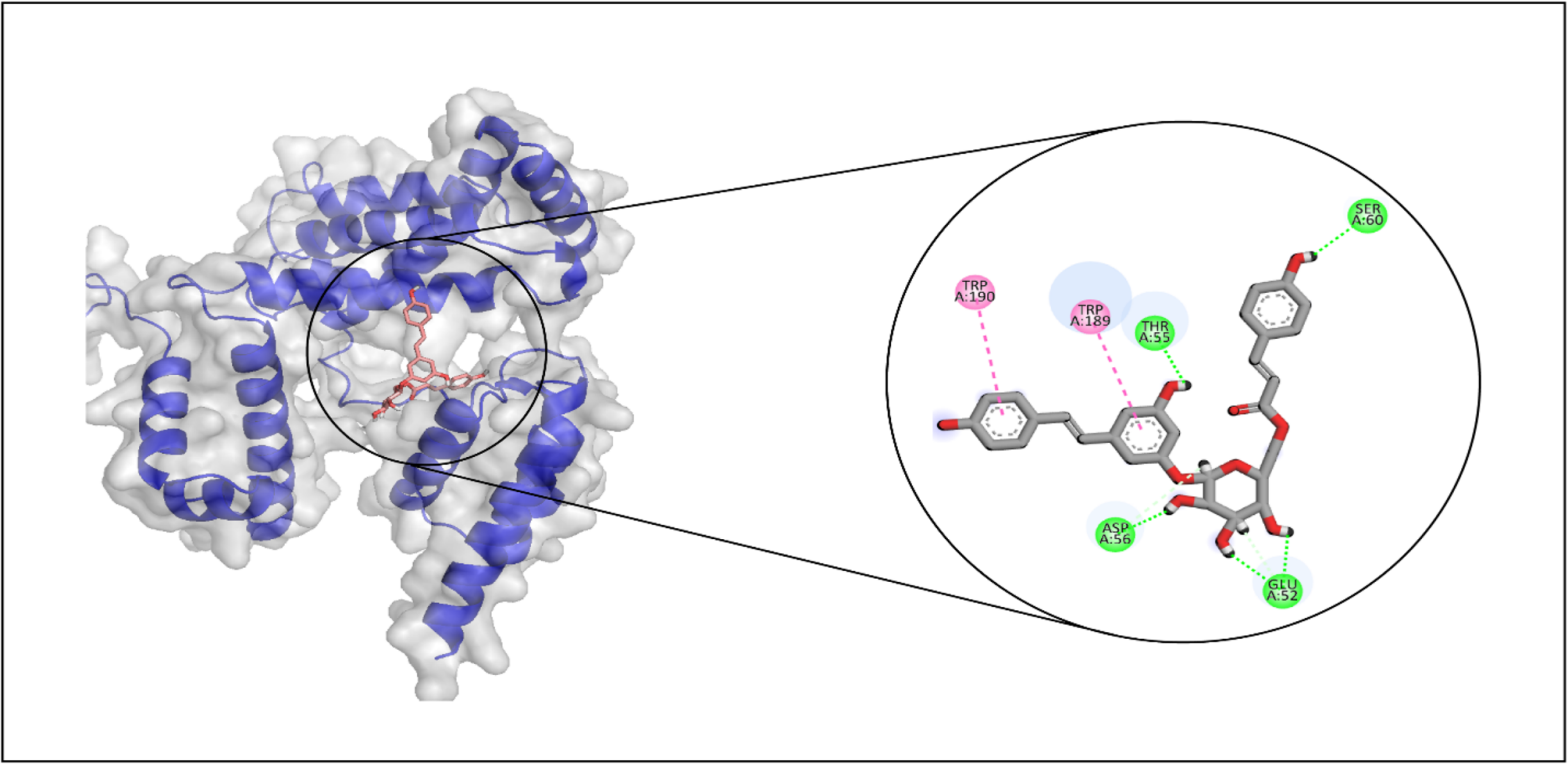
The molecular interactions of LTS0236112 against RpoD. The magenta spheres (hydrophobic/pi-alkyl) and the green spheres (hydrogen bonds) represent the molecular interactions.

Among RpoD binding affinities, LTS0236112 exhibited the highest value. The molecular interactions showed that LTS0236112 made four hydrogen bonds with Thr55, Ser60, Asp56, and Glu52, and two hydrophobic interactions with Trp189 and Trp190 as shown in Fig. 9.

**Figure 10:**
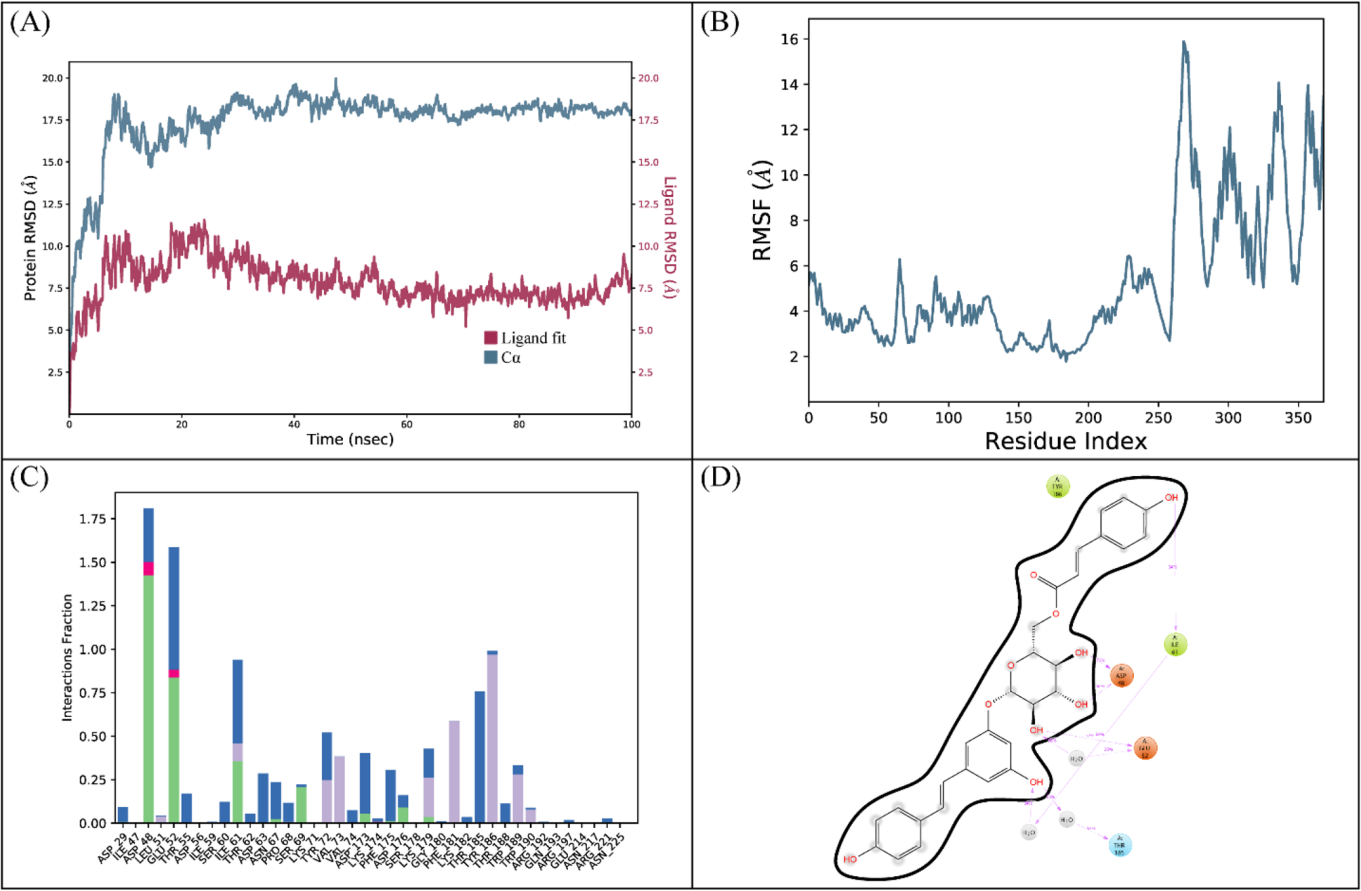
The MD trajectory analysis of the RpoD complex. (**A**) RMSD of C-alpha atoms of proteins and ligand atoms fit on protein. (**B**) RMSF analysis. (**C**) Protein-ligand contacts calculated during simulation. (**D**) Tendency of the interacting residues with the ligand during simulation.

The protein’s C-alpha atoms’ RMSD progressively grew to approximately 17.5 at 20 ns due to the presence of large loops in the protein structure. The protein structure equilibrated at the 20 ns and then it did not show major deviations which indicated that after 20 ns, the protein structure attained stable confirmation as the RMSD stayed in the range of 17–17.5 Å until the simulation’s ending. The RMSD of ligand was lower than the protein atoms (Fig. 10A). The RMSF analysis showed that that most of the residues showed high fluctuations, especially the residues ranging from 250 to 350 due to the presence of large loop regions (Fig. 10B). In protein-ligand contact analysis, the residues involved in hydrogen bonding were Asp48, Glu52, Ile61, Ser69, and Asp176. Asp48 and Glu52 were also involved in ionic interactions during the simulation (Fig. 10C). Asp48 showed the strongest propensity for binding among these interacting residues, with interactions being seen in 73% of the total frames (Fig. 10D).

The binding free energy calculation showed that the total binding free energy of the complex was −51.35 kcal/mol. The other energy components are shown in Table 7.

**Table 7:** The contribution of the energy components in total binding free energy in RpoD complex.

Components	Binding energy (kcal/mol)
Bind	−51.35
Coulomb	−22.70
Covalent	−0.71
Solv GB	−32.48
vdW	−32.19

## Discussion

*Streptococcus gallolyticus*, which can cause bacterial endocarditis in up to 15–20% of cases, is one of the most frequent causes of the condition. People with a history of intravenous drug use, heart valve disease, or congenital heart defects are more likely to develop Sg endocarditis. Sg-induced endocarditis can be a fatal and hazardous condition, with a mortality rate ranging from 10 to 50%. Bacteria can enter the bloodstream via the mouth, gastrointestinal or oral surgery, or other sources of infection. Antibiotic resistance is a known complication of Sg that can make treatment more difficult or even result in treatment failure^35^. Therefore, a drug that effectively combats Sg must be created.

Core proteomics along with the subtractive proteomics approach was used in our investigation to screen therapeutic candidates against Sg. This method identifies targets by identifying essential and nonhomologous proteins inside pathogenic organisms. This approach ensures that the targets are essential for bacterial survival, increasing the chances of developing effective antibacterial agents. By excluding any proteins with human homologs, subtractive proteomics significantly reduces the risk of cross-reactivity and potential side effects, thereby improving the safety profile of the drugs produced. This strategic method directs drug development efforts toward bacterial-specific proteins, resulting in greater specificity and efficacy in treating bacterial infections^4–6^. Therapeutic target identification is a critical step in computer-based drug design methods^36^. Recent developments in bioinformatics and computational biology have led to a variety of drug design and in silico analysis techniques that shorten the time and expense involved in the trial and error of ions used in drug development^37^.

The core proteomes of seven renowned pathogenic Sg strains were analyzed in order to evaluate them for drug development. In drug development, targeting core proteins in pathogens can be a viable method, particularly for infectious disorders. It has the potential for broad-spectrum action as well as decreased resistance development. Hence, 9576 proteins were retrieved from core proteome analysis. Bacterial essential gene research contributes to a better understanding of the nature of life and the discovery of new therapeutic targets for treating pathogenic illnesses. The development of vaccines and antibacterial drugs preferentially targets essential proteins^38,39^. A subtractive proteomics process was applied to the core proteome, discovering 325 essential proteins. These proteins could be human homologs. Targeting these proteins can therefore have a devastating impact on human metabolism. The use of non-homologous proteins can help to reduce the possibility of cross-reactivity as well as other undesired effects^37^. To avoid such unfavorable conditions and toxicity, 138 non-homologous proteins were screened.

Predicting the subcellular location of a protein is an easy and inexpensive way to determine its function^40^. Cytoplasmic proteins are more appealing as therapeutic targets because membrane-based proteins are more challenging to purify and study^41^. Hence, 109 cytoplasmic proteins were screened. These proteins were then evaluated for their virulence factor and 12 proteins were found to be virulent. Further, druggability of virulent proteins was analyzed for all drug targets in the DrugBank database and ten druggable proteins were found. Having a detailed understanding of biological mechanisms, molecular activities, and the particulars of protein structure is essential to the entire drug development process. Hence, three proteins were identified as potential drug targets on the basis of functional prediction: glucosamine-1phosphate N-acetyltransferase (GlmU), RNA polymerase sigma factor (RpoD), and pantetheine-phosphate adenylyltransferase (PPAT).

3D structures of the target protein were predicted through Swiss Model. A natural product library containing 10,000 molecules from the LOTUS database was docked against therapeutic target proteins. Molecular docking is a method of predicting the orientation of tiny molecules in relation to their protein targets^42^. The best 10 compounds that docked against each target protein according to the glide gscore were selected.

Further, the ADMET analysis of the compounds were performed. The ADMET analysis revealed that most of the compounds violated the Lipinski rule of five due to their large molecular weights. However, natural products have unique chemical scaffolds: natural products often possess complex and unique chemical scaffolds that cannot be easily replicated by synthetic chemistry. These scaffolds may exhibit desirable biological activities despite violating Lipinski's rules^7^. Several approved drugs derived from natural products, such as atorvastatin, montelukast, and paclitaxel, violate Lipinski's rules yet demonstrate oral bioavailability and therapeutic efficacy. These examples validate the potential of larger, more complex natural products as drug candidates^8^.

To identify their molecular interactions, the binding poses of the substances with the highest binding affinities to the target proteins were examined. LTS0001632 had the greatest binding affinity for GlmU, LTS0243441 had the greatest binding affinity for PPAT, and LTS0236112 had the greatest binding affinity for RpoD. These compounds formed hydrogen bonds with their respective protein residues. The fact that these compounds have hydrogen bonds with residues suggests that they are positioned correctly within the active site, effectively interacting with essential functional groups that are crucial to the activity of the protein. In addition to hydrogen bonds, compounds also made hydrophobic interactions, which further stabilized the inhibitor within the binding site. Hydrophobic interactions, typically involving non-polar side chains, contribute to the overall binding affinity by providing additional attractive forces that complement hydrogen bonding. These interactions were primarily with hydrophobic residues surrounding the binding pocket, enhancing the compound's stability and binding efficacy^9,10^.

Because these ligands exhibited a robust molecular interaction network and a high dock score, the best docked complexes were submitted to MMGBSA and molecular dynamics (MD) simulations. The dynamics, stability, and binding affinity of protein-ligand interactions can be investigated using MD simulation and MMGBSA analysis of docked complexes^43^. After analyzing the MD simulation results, it was discovered that all of the complexes maintained good stability throughout the simulation. The RMSD value of the complexes indicates good structural stability and strong intramolecular interaction with residues throughout the simulation period. RMSF aids in the identification of flexible and rigid regions in proteins, thereby improving our understanding of protein function and interactions. According to RMSF analysis, in GImu- LTS0001632 complex and PPAT -LTS0243441 complex most of the residues remained rigid throughout the simulation. Although RpoD-LTS0236112 complex showed slightly higher residue fluctuation as compared to the other two complexes. Protein-ligand contacts are one of the most important aspects of molecular interactions in biological systems. These contacts lay the groundwork for molecular recognition and selectivity by providing directionality and explicitness to molecular interactions. Hence, MD simulation results ensured that the selected inhibitors bind effectively and remain stable within the active site, indicating their potential efficacy in a biological context.

Protein and ligand binding free energies are very good predictors of their interactions. The binding free energy was calculated by adding the Coulomb, Van der Waals, Solvation, and Covalent energies together. The electrostatic interactions between charged particles in a system are represented by Coulomb energy. Van der Waals energy describes the non-covalent interactions that result from induced dipoles in molecules. Solvation energy is a combination of polar solvation energy (estimated using the Generalized Born (GB) model) and nonpolar solvation energy (typically represented by a Surface Area (SA) term). Covalent energy includes bonded interactions such as bond stretching, angle bending, and torsional angles within molecules^11^. Total binding free energies of the GImu-LTS0001632, PPAT-LTS0243441, and RpoD-LTS0236112 were −83.52, -67.66, and −51.35 kcal/mol. GImu-LTS0001632 has the highest binding energy of the three complexes, indicating that it is more stable and the interaction is more energetically favorable.

Hence, our work integrates these computational techniques in a novel way into a coherent pipeline, which simplifies the entire drug discovery process, from target identification to inhibitor validation. This all-encompassing strategy not only raises the bar for computational drug discovery in bacterial pathogens but also speeds up the identification of new drug targets and inhibitors. The results offer a solid foundation for further investigation and advancement, which may result in the development of novel antibiotics that are safe and effective in treating the serious problem of antibiotic resistance in *S. gallolyticus*.

## Conclusion

Recognizing the importance of identifying effective pharmaceutical targets, the purpose of this study was to undertake a computational assessment of the human microbial pathogen *Streptococcus gallolyticus* to identify potential targets using a variety of computational software and techniques. Based on subcellular localization and druggability, three proteins were identified as drug targets in the initial phase of the study. The structural analysis of potential targets was carried out in the second phase, which was then used for molecular docking and simulation experiments. Therefore, this work marks a substantial step forward in the creation of fresh, effective anti-*S. gallolyticus* drugs.

### Supplementary Information

Supplementary Table S1.

## Data Availability

We didnot produce any data in this study. But used the already submitted data from National Center for Biotechnology Information (NCBI) database for data analysis. The data used in this study has the following accession numbers in NCBI database. GCA_013267695.1 GCA_027474865.2 GCA_001477575.1 GCA_000027185.1 GCA_000270145.1 GCA_000203195.1 GCA_019021805.1.
